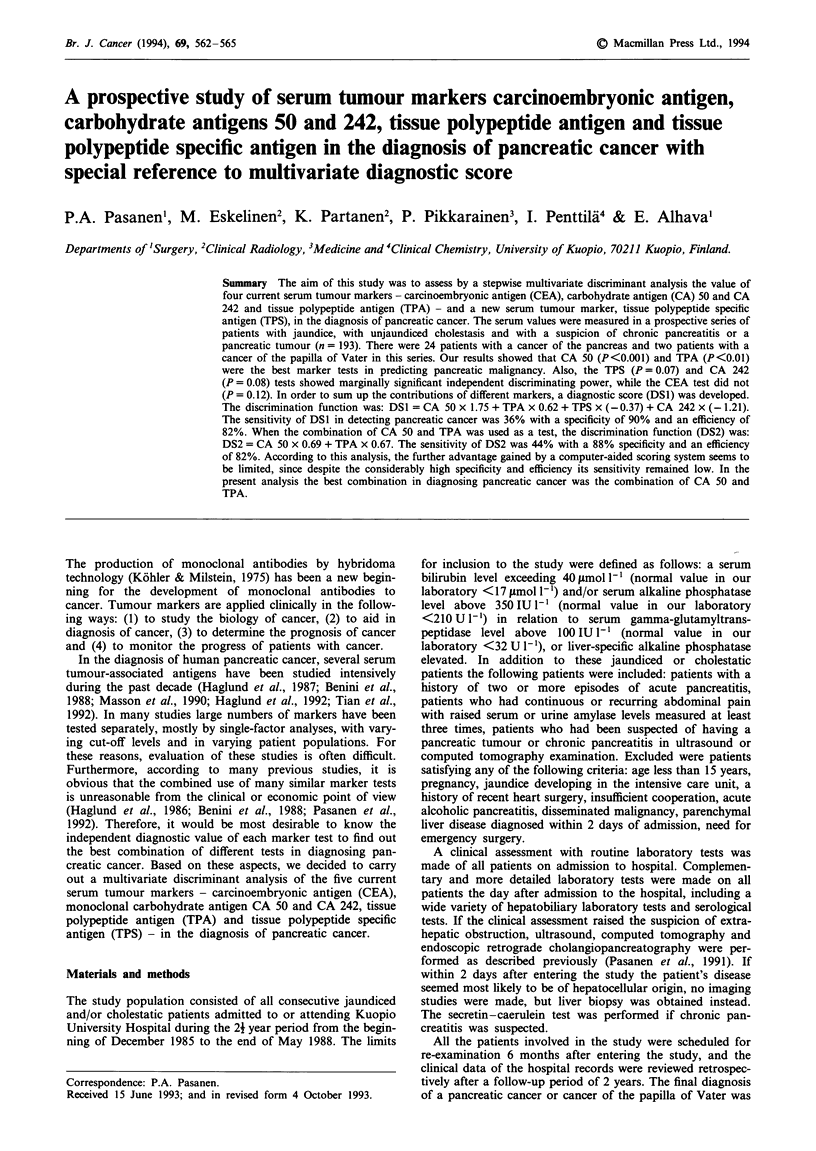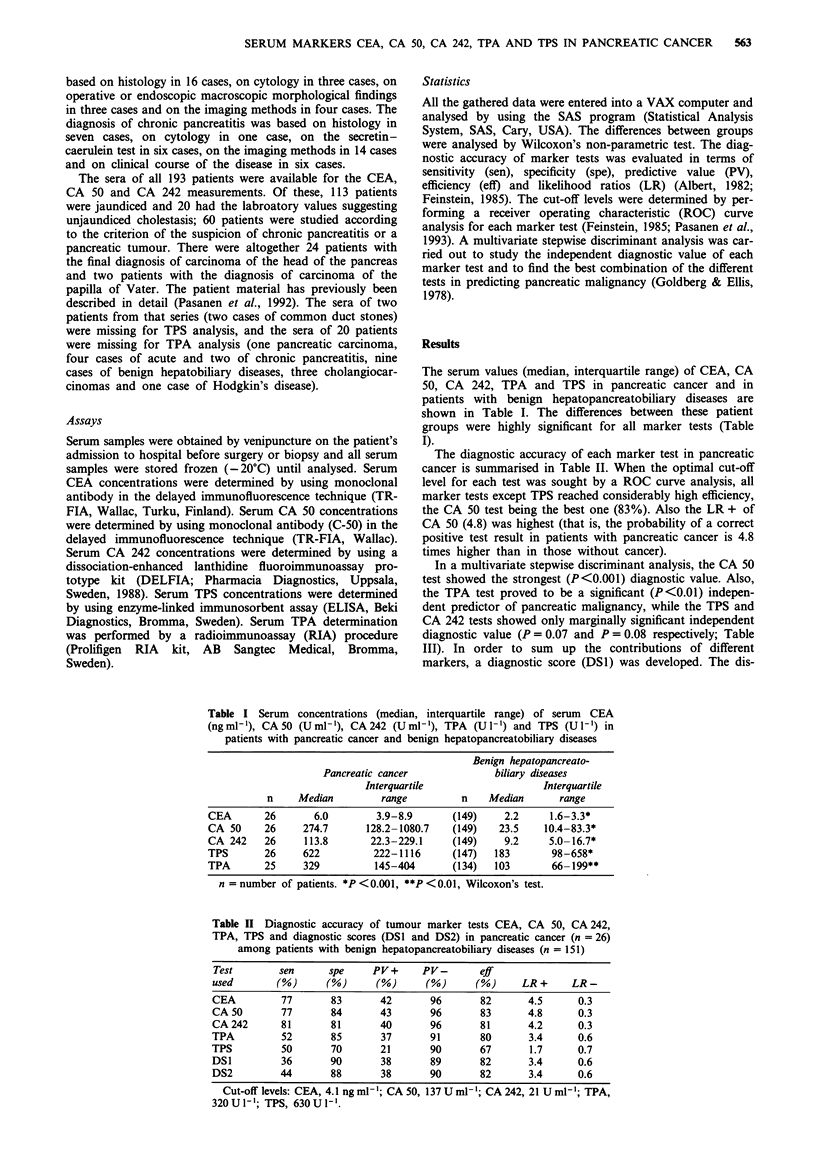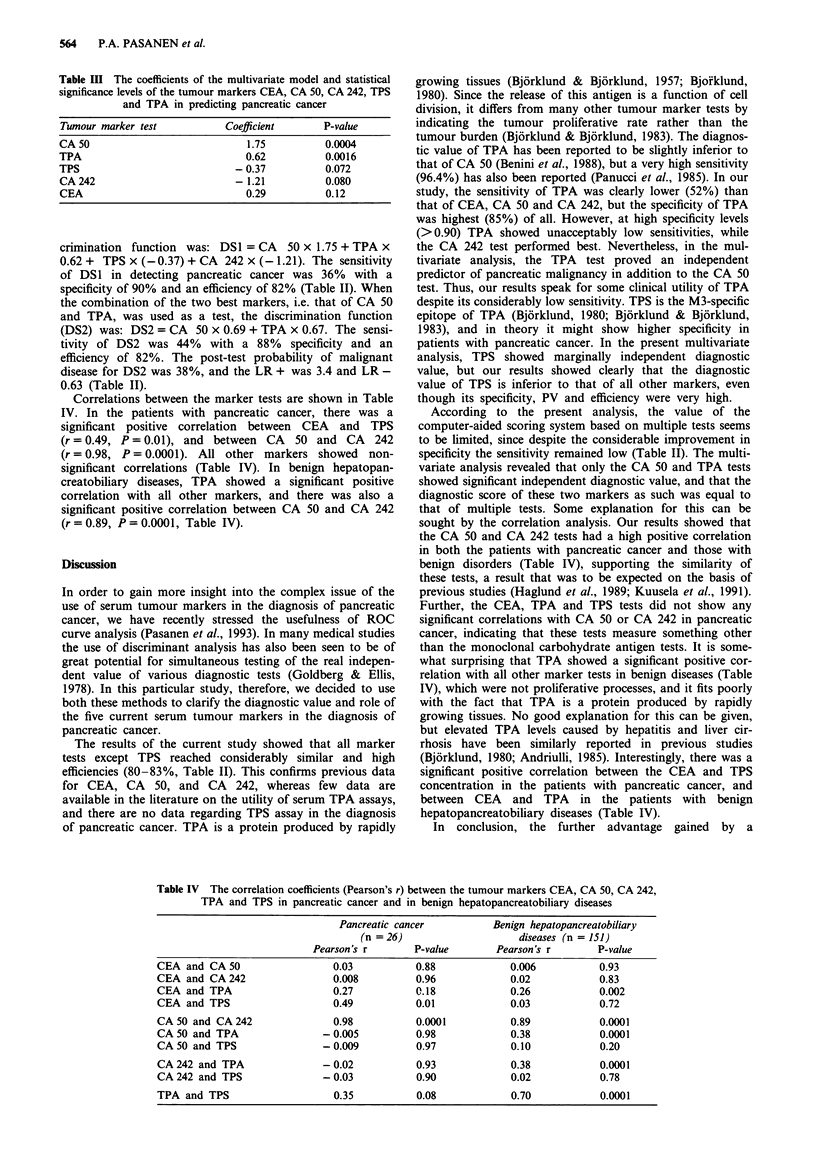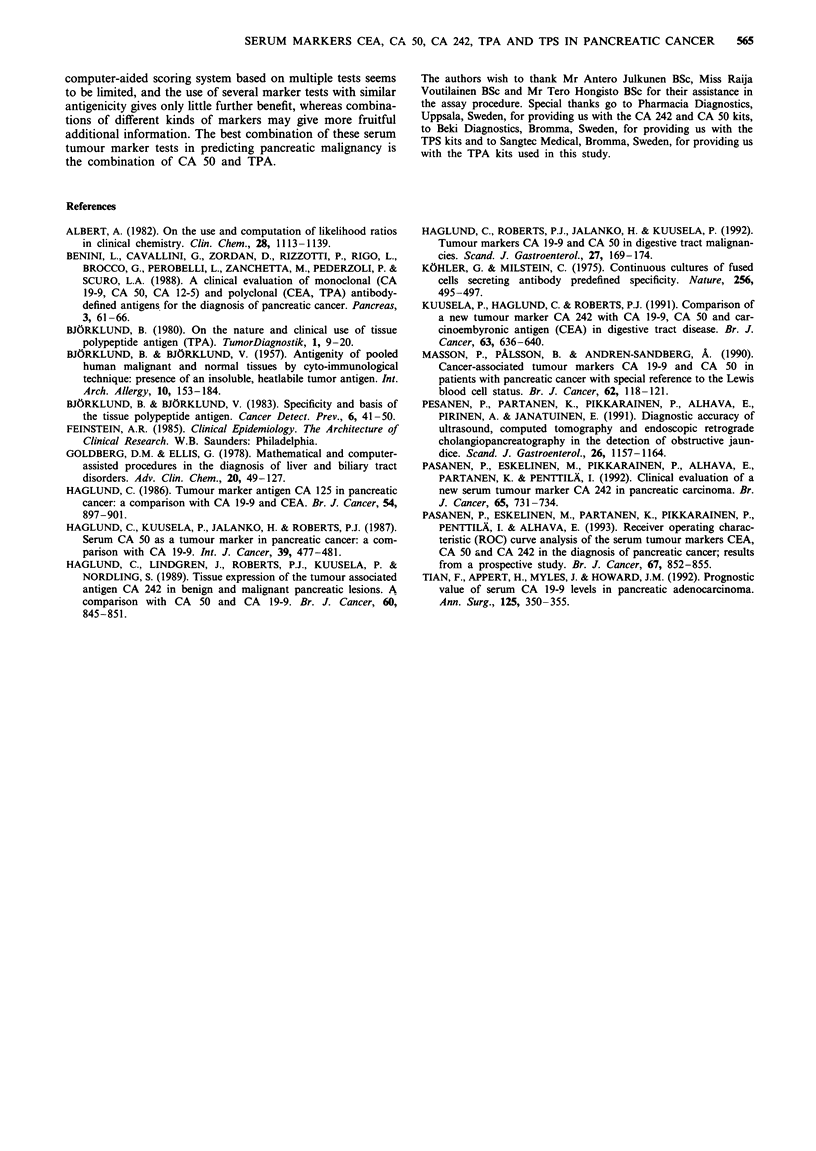# A prospective study of serum tumour markers carcinoembryonic antigen, carbohydrate antigens 50 and 242, tissue polypeptide antigen and tissue polypeptide specific antigen in the diagnosis of pancreatic cancer with special reference to multivariate diagnostic score.

**DOI:** 10.1038/bjc.1994.102

**Published:** 1994-03

**Authors:** P. A. Pasanen, M. Eskelinen, K. Partanen, P. Pikkarainen, I. Penttilä, E. Alhava

**Affiliations:** Department of Surgery, University of Kuopio, Finland.

## Abstract

The aim of this study was to assess by a stepwise multivariate discriminant analysis the value of four current serum tumour markers - carcinoembryonic antigen (CEA), carbohydrate antigen (CA) 50 and CA 242 and tissue polypeptide antigen (TPA) - and a new serum tumour marker, tissue polypeptide specific antigen (TPS), in the diagnosis of pancreatic cancer. The serum values were measured in a prospective series of patients with jaundice, with unjaundiced cholestasis and with a suspicion of chronic pancreatitis or a pancreatic tumour (n = 193). There were 24 patients with a cancer of the pancreas and two patients with a cancer of the papilla of Vater in this series. Our results showed that CA 50 (P < 0.001) and TPA (P < 0.01) were the best marker tests in predicting pancreatic malignancy. Also, the TPS (P = 0.07) and CA 242 (P = 0.08) tests showed marginally significant independent discriminating power, while the CEA test did not (P = 0.12). In order to sum up the contributions of different markers, a diagnostic score (DSI) was developed. The discrimination function was: DS1 = CA 50 x 1.75 + TPA x 0.62 + TPS x (-0.37) + CA 242 x (-1.21). The sensitivity of DS1 in detecting pancreatic cancer was 36% with a specificity of 90% and an efficiency of 82%. When the combination of CA 50 and TPA was used as a test, the discrimination function (DS2) was: DS2 = CA 50 x 0.69 + TPA x 0.67. The sensitivity of DS2 was 44% with a 88% specificity and an efficiency of 82%. According to this analysis, the further advantage gained by a computer-aided scoring system seems to be limited, since despite the considerably high specificity and efficiency its sensitivity remained low. In the present analysis the best combination in diagnosing pancreatic cancer was the combination of CA 50 and TPA.


Br. J. Cancer (1994), 69, 562-565 © Macmillan Press Ltd., 1994

### A prospective study of serum tumour markers carcinoembryonic antigen, carbohydrate antigens 50 and 242, tissue polypeptide antigen and tissue polypeptide specific antigen in the diagnosis of pancreatic cancer with special reference to multivariate diagnostic score

P.A. Pasanen[1], M. Eskelinen[2], K. Partanen[2], P. Pikkarainen[3], I. Penttilä[4] & E. Alhava[1]

*Departments of [1]Surgery, [2]Clinical Radiology, [3]Medicine and [4]Clinical Chemistry, University of Kuopio, 70211 Kuopio, Finland.*

**Summary** The aim of this study was to assess by a stepwise multivariate discriminant analysis the value of four current serum tumour markers – carcinoembryonic antigen (CEA), carbohydrate antigen (CA) 50 and CA 242 and tissue polypeptide antigen (TPA) – and a new serum tumour marker, tissue polypeptide specific antigen (TPS), in the diagnosis of pancreatic cancer. The serum values were measured in a prospective series of patients with jaundice, with unjaundiced cholestasis and with a suspicion of chronic pancreatitis or a pancreatic tumour ($n = 193$). There were 24 patients with a cancer of the pancreas and two patients with a cancer of the papilla of Vater in this series. Our results showed that CA 50 ($P < 0.001$) and TPA ($P < 0.01$) were the best marker tests in predicting pancreatic malignancy. Also, the TPS ($P = 0.07$) and CA 242 ($P = 0.08$) tests showed marginally significant independent discriminating power, while the CEA test did not ($P = 0.12$). In order to sum up the contributions of different markers, a diagnostic score (DS1) was developed. The discrimination function was: DS1 = CA 50 × 1.75 + TPA × 0.62 + TPS × (−0.37) + CA 242 × (−1.21). The sensitivity of DS1 in detecting pancreatic cancer was 36% with a specificity of 90% and an efficiency of 82%. When the combination of CA 50 and TPA was used as a test, the discrimination function (DS2) was: DS2 = CA 50 × 0.69 + TPA × 0.67. The sensitivity of DS2 was 44% with a 88% specificity and an efficiency of 82%. According to this analysis, the further advantage gained by a computer-aided scoring system seems to be limited, since despite the considerably high specificity and efficiency its sensitivity remained low. In the present analysis the best combination in diagnosing pancreatic cancer was the combination of CA 50 and TPA.

The production of monoclonal antibodies by hybridoma technology (Köhler & Milstein, 1975) has been a new beginning for the development of monoclonal antibodies to cancer. Tumour markers are applied clinically in the following ways: (1) to study the biology of cancer, (2) to aid in diagnosis of cancer, (3) to determine the prognosis of cancer and (4) to monitor the progress of patients with cancer.

In the diagnosis of human pancreatic cancer, several serum tumour-associated antigens have been studied intensively during the past decade (Haglund *et al.*, 1987; Benini *et al.*, 1988; Masson *et al.*, 1990; Haglund *et al.*, 1992; Tian *et al.*, 1992). In many studies large numbers of markers have been tested separately, mostly by single-factor analyses, with varying cut-off levels and in varying patient populations. For these reasons, evaluation of these studies is often difficult. Furthermore, according to many previous studies, it is obvious that the combined use of many similar marker tests is unreasonable from the clinical or economic point of view (Haglund *et al.*, 1986; Benini *et al.*, 1988; Pasanen *et al.*, 1992). Therefore, it would be most desirable to know the independent diagnostic value of each marker test to find out the best combination of different tests in diagnosing pancreatic cancer. Based on these aspects, we decided to carry out a multivariate discriminant analysis of the five current serum tumour markers – carcinoembryonic antigen (CEA), monoclonal carbohydrate antigen CA 50 and CA 242, tissue polypeptide antigen (TPA) and tissue polypeptide specific antigen (TPS) – in the diagnosis of pancreatic cancer.

### Materials and methods

The study population consisted of all consecutive jaundiced and/or cholestatic patients admitted to or attending Kuopio University Hospital during the 2½ year period from the beginning of December 1985 to the end of May 1988. The limits for inclusion to the study were defined as follows: a serum bilirubin level exceeding 40 $\mu mol\ l^{-1}$ (normal value in our laboratory <17 $\mu mol\ l^{-1}$) and/or serum alkaline phosphatase level above 350 $IU\ l^{-1}$ (normal value in our laboratory <210 $U\ l^{-1}$) in relation to serum gamma-glutamyltranspeptidase level above 100 $IU\ l^{-1}$ (normal value in our laboratory <32 $U\ l^{-1}$), or liver-specific alkaline phosphatase elevated. In addition to these jaundiced or cholestatic patients the following patients were included: patients with a history of two or more episodes of acute pancreatitis, patients who had continuous or recurring abdominal pain with raised serum or urine amylase levels measured at least three times, patients who had been suspected of having a pancreatic tumour or chronic pancreatitis in ultrasound or computed tomography examination. Excluded were patients satisfying any of the following criteria: age less than 15 years, pregnancy, jaundice developing in the intensive care unit, a history of recent heart surgery, insufficient cooperation, acute alcoholic pancreatitis, disseminated malignancy, parenchymal liver disease diagnosed within 2 days of admission, need for emergency surgery.

A clinical assessment with routine laboratory tests was made of all patients on admission to hospital. Complementary and more detailed laboratory tests were made on all patients the day after admission to the hospital, including a wide variety of hepatobiliary laboratory tests and serological tests. If the clinical assessment raised the suspicion of extrahepatic obstruction, ultrasound, computed tomography and endoscopic retrograde cholangiopancreatography were performed as described previously (Pasanen *et al.*, 1991). If within 2 days after entering the study the patient's disease seemed most likely to be of hepatocellular origin, no imaging studies were made, but liver biopsy was obtained instead. The secretin–caerulein test was performed if chronic pancreatitis was suspected.

All the patients involved in the study were scheduled for re-examination 6 months after entering the study, and the clinical data of the hospital records were reviewed retrospectively after a follow-up period of 2 years. The final diagnosis of a pancreatic cancer or cancer of the papilla of Vater was

Correspondence: P.A. Pasanen.
Received 15 June 1993; and in revised form 4 October 1993.

based on histology in 16 cases, on cytology in three cases, on operative or endoscopic macroscopic morphological findings in three cases and on the imaging methods in four cases. The diagnosis of chronic pancreatitis was based on histology in seven cases, on cytology in one case, on the secretin–caerulein test in six cases, on the imaging methods in 14 cases and on clinical course of the disease in six cases.

The sera of all 193 patients were available for the CEA, CA 50 and CA 242 measurements. Of these, 113 patients were jaundiced and 20 had the labroatory values suggesting unjaundiced cholestasis; 60 patients were studied according to the criterion of the suspicion of chronic pancreatitis or a pancreatic tumour. There were altogether 24 patients with the final diagnosis of carcinoma of the head of the pancreas and two patients with the diagnosis of carcinoma of the papilla of Vater. The patient material has previously been described in detail (Pasanen *et al.*, 1992). The sera of two patients from that series (two cases of common duct stones) were missing for TPS analysis, and the sera of 20 patients were missing for TPA analysis (one pancreatic carcinoma, four cases of acute and two of chronic pancreatitis, nine cases of benign hepatobiliary diseases, three cholangiocarcinomas and one case of Hodgkin's disease).

*Assays*

Serum samples were obtained by venipuncture on the patient's admission to hospital before surgery or biopsy and all serum samples were stored frozen (−20°C) until analysed. Serum CEA concentrations were determined by using monoclonal antibody in the delayed immunofluorescence technique (TR-FIA, Wallac, Turku, Finland). Serum CA 50 concentrations were determined by using monoclonal antibody (C-50) in the delayed immunofluorescence technique (TR-FIA, Wallac). Serum CA 242 concentrations were determined by using a dissociation-enhanced lanthidine fluoroimmunoassay prototype kit (DELFIA; Pharmacia Diagnostics, Uppsala, Sweden, 1988). Serum TPS concentrations were determined by using enzyme-linked immunosorbent assay (ELISA, Beki Diagnostics, Bromma, Sweden). Serum TPA determination was performed by a radioimmunoassay (RIA) procedure (Prolifigen RIA kit, AB Sangtec Medical, Bromma, Sweden).

*Statistics*

All the gathered data were entered into a VAX computer and analysed by using the SAS program (Statistical Analysis System, SAS, Cary, USA). The differences between groups were analysed by Wilcoxon's non-parametric test. The diagnostic accuracy of marker tests was evaluated in terms of sensitivity (sen), specificity (spe), predictive value (PV), efficiency (eff) and likelihood ratios (LR) (Albert, 1982; Feinstein, 1985). The cut-off levels were determined by performing a receiver operating characteristic (ROC) curve analysis for each marker test (Feinstein, 1985; Pasanen *et al.*, 1993). A multivariate stepwise discriminant analysis was carried out to study the independent diagnostic value of each marker test and to find the best combination of the different tests in predicting pancreatic malignancy (Goldberg & Ellis, 1978).

### Results

The serum values (median, interquartile range) of CEA, CA 50, CA 242, TPA and TPS in pancreatic cancer and in patients with benign hepatopancreatobiliary diseases are shown in Table I. The differences between these patient groups were highly significant for all marker tests (Table I).

The diagnostic accuracy of each marker test in pancreatic cancer is summarised in Table II. When the optimal cut-off level for each test was sought by a ROC curve analysis, all marker tests except TPS reached considerably high efficiency, the CA 50 test being the best one (83%). Also the LR + of CA 50 (4.8) was highest (that is, the probability of a correct positive test result in patients with pancreatic cancer is 4.8 times higher than in those without cancer).

In a multivariate stepwise discriminant analysis, the CA 50 test showed the strongest ($P<0.001$) diagnostic value. Also, the TPA test proved to be a significant ($P<0.01$) independent predictor of pancreatic malignancy, while the TPS and CA 242 tests showed only marginally significant independent diagnostic value ($P=0.07$ and $P=0.08$ respectively; Table III). In order to sum up the contributions of different markers, a diagnostic score (DS1) was developed. The dis-

**Table I** Serum concentrations (median, interquartile range) of serum CEA (ng ml$^{-1}$), CA 50 (U ml$^{-1}$), CA 242 (U ml$^{-1}$), TPA (U l$^{-1}$) and TPS (U l$^{-1}$) in patients with pancreatic cancer and benign hepatopancreatobiliary diseases

| | *Pancreatic cancer* | | | *Benign hepatopancreatobiliary diseases* | | |
|---|---|---|---|---|---|---|
| | n | *Median* | *Interquartile range* | n | *Median* | *Interquartile range* |
| CEA | 26 | 6.0 | 3.9–8.9 | (149) | 2.2 | 1.6–3.3* |
| CA 50 | 26 | 274.7 | 128.2–1080.7 | (149) | 23.5 | 10.4–83.3* |
| CA 242 | 26 | 113.8 | 22.3–229.1 | (149) | 9.2 | 5.0–16.7* |
| TPS | 26 | 622 | 222–1116 | (147) | 183 | 98–658* |
| TPA | 25 | 329 | 145–404 | (134) | 103 | 66–199** |

*n* = number of patients. *$P<0.001$, **$P<0.01$, Wilcoxon's test.

**Table II** Diagnostic accuracy of tumour marker tests CEA, CA 50, CA 242, TPA, TPS and diagnostic scores (DS1 and DS2) in pancreatic cancer ($n=26$) among patients with benign hepatopancreatobiliary diseases ($n=151$)

| *Test used* | *sen (%)* | *spe (%)* | *PV+ (%)* | *PV− (%)* | *eff (%)* | *LR+* | *LR−* |
|---|---|---|---|---|---|---|---|
| CEA | 77 | 83 | 42 | 96 | 82 | 4.5 | 0.3 |
| CA 50 | 77 | 84 | 43 | 96 | 83 | 4.8 | 0.3 |
| CA 242 | 81 | 81 | 40 | 96 | 81 | 4.2 | 0.3 |
| TPA | 52 | 85 | 37 | 91 | 80 | 3.4 | 0.6 |
| TPS | 50 | 70 | 21 | 90 | 67 | 1.7 | 0.7 |
| DS1 | 36 | 90 | 38 | 89 | 82 | 3.4 | 0.6 |
| DS2 | 44 | 88 | 38 | 90 | 82 | 3.4 | 0.6 |

Cut-off levels: CEA, 4.1 ng ml$^{-1}$; CA 50, 137 U ml$^{-1}$; CA 242, 21 U ml$^{-1}$; TPA, 320 U l$^{-1}$; TPS, 630 U l$^{-1}$.

**Table III** The coefficients of the multivariate model and statistical significance levels of the tumour markers CEA, CA 50, CA 242, TPS and TPA in predicting pancreatic cancer

| *Tumour marker test* | *Coefficient* | P-*value* |
|---|---|---|
| CA 50 | 1.75 | 0.0004 |
| TPA | 0.62 | 0.0016 |
| TPS | − 0.37 | 0.072 |
| CA 242 | − 1.21 | 0.080 |
| CEA | 0.29 | 0.12 |

crimination function was: DS1 = CA 50 × 1.75 + TPA × 0.62 + TPS × (−0.37) + CA 242 × (−1.21). The sensitivity of DS1 in detecting pancreatic cancer was 36% with a specificity of 90% and an efficiency of 82% (Table II). When the combination of the two best markers, i.e. that of CA 50 and TPA, was used as a test, the discrimination function (DS2) was: DS2 = CA 50 × 0.69 + TPA × 0.67. The sensitivity of DS2 was 44% with a 88% specificity and an efficiency of 82%. The post-test probability of malignant disease for DS2 was 38%, and the LR + was 3.4 and LR − 0.63 (Table II).

Correlations between the marker tests are shown in Table IV. In the patients with pancreatic cancer, there was a significant positive correlation between CEA and TPS ($r = 0.49$, $P = 0.01$), and between CA 50 and CA 242 ($r = 0.98$, $P = 0.0001$). All other markers showed non-significant correlations (Table IV). In benign hepatopancreatobiliary diseases, TPA showed a significant positive correlation with all other markers, and there was also a significant positive correlation between CA 50 and CA 242 ($r = 0.89$, $P = 0.0001$, Table IV).

### Discussion

In order to gain more insight into the complex issue of the use of serum tumour markers in the diagnosis of pancreatic cancer, we have recently stressed the usefulness of ROC curve analysis (Pasanen *et al.*, 1993). In many medical studies the use of discriminant analysis has also been seen to be of great potential for simultaneous testing of the real independent value of various diagnostic tests (Goldberg & Ellis, 1978). In this particular study, therefore, we decided to use both these methods to clarify the diagnostic value and role of the five current serum tumour markers in the diagnosis of pancreatic cancer.

The results of the current study showed that all marker tests except TPS reached considerably similar and high efficiencies (80–83%, Table II). This confirms previous data for CEA, CA 50, and CA 242, whereas few data are available in the literature on the utility of serum TPA assays, and there are no data regarding TPS assay in the diagnosis of pancreatic cancer. TPA is a protein produced by rapidly growing tissues (Björklund & Björklund, 1957; Bjořklund, 1980). Since the release of this antigen is a function of cell division, it differs from many other tumour marker tests by indicating the tumour proliferative rate rather than the tumour burden (Björklund & Björklund, 1983). The diagnostic value of TPA has been reported to be slightly inferior to that of CA 50 (Benini *et al.*, 1988), but a very high sensitivity (96.4%) has also been reported (Panucci *et al.*, 1985). In our study, the sensitivity of TPA was clearly lower (52%) than that of CEA, CA 50 and CA 242, but the specificity of TPA was highest (85%) of all. However, at high specificity levels (>0.90) TPA showed unacceptably low sensitivities, while the CA 242 test performed best. Nevertheless, in the multivariate analysis, the TPA test proved an independent predictor of pancreatic malignancy in addition to the CA 50 test. Thus, our results speak for some clinical utility of TPA despite its considerably low sensitivity. TPS is the M3-specific epitope of TPA (Björklund, 1980; Björklund & Björklund, 1983), and in theory it might show higher specificity in patients with pancreatic cancer. In the present multivariate analysis, TPS showed marginally independent diagnostic value, but our results showed clearly that the diagnostic value of TPS is inferior to that of all other markers, even though its specificity, PV and efficiency were very high.

According to the present analysis, the value of the computer-aided scoring system based on multiple tests seems to be limited, since despite the considerable improvement in specificity the sensitivity remained low (Table II). The multivariate analysis revealed that only the CA 50 and TPA tests showed significant independent diagnostic value, and that the diagnostic score of these two markers as such was equal to that of multiple tests. Some explanation for this can be sought by the correlation analysis. Our results showed that the CA 50 and CA 242 tests had a high positive correlation in both the patients with pancreatic cancer and those with benign disorders (Table IV), supporting the similarity of these tests, a result that was to be expected on the basis of previous studies (Haglund *et al.*, 1989; Kuusela *et al.*, 1991). Further, the CEA, TPA and TPS tests did not show any significant correlations with CA 50 or CA 242 in pancreatic cancer, indicating that these tests measure something other than the monoclonal carbohydrate antigen tests. It is somewhat surprising that TPA showed a significant positive correlation with all other marker tests in benign diseases (Table IV), which were not proliferative processes, and it fits poorly with the fact that TPA is a protein produced by rapidly growing tissues. No good explanation for this can be given, but elevated TPA levels caused by hepatitis and liver cirrhosis have been similarly reported in previous studies (Björklund, 1980; Andriulli, 1985). Interestingly, there was a significant positive correlation between the CEA and TPS concentration in the patients with pancreatic cancer, and between CEA and TPA in the patients with benign hepatopancreatobiliary diseases (Table IV).

In conclusion, the further advantage gained by a

**Table IV** The correlation coefficients (Pearson's *r*) between the tumour markers CEA, CA 50, CA 242, TPA and TPS in pancreatic cancer and in benign hepatopancreatobiliary diseases

| | *Pancreatic cancer (*n *= 26)* | | *Benign hepatopancreatobiliary diseases (*n *= 151)* | |
|---|---|---|---|---|
| | *Pearson's* r | P-*value* | *Pearson's* r | P-*value* |
| CEA and CA 50 | 0.03 | 0.88 | 0.006 | 0.93 |
| CEA and CA 242 | 0.008 | 0.96 | 0.02 | 0.83 |
| CEA and TPA | 0.27 | 0.18 | 0.26 | 0.002 |
| CEA and TPS | 0.49 | 0.01 | 0.03 | 0.72 |
| CA 50 and CA 242 | 0.98 | 0.0001 | 0.89 | 0.0001 |
| CA 50 and TPA | − 0.005 | 0.98 | 0.38 | 0.0001 |
| CA 50 and TPS | − 0.009 | 0.97 | 0.10 | 0.20 |
| CA 242 and TPA | − 0.02 | 0.93 | 0.38 | 0.0001 |
| CA 242 and TPS | − 0.03 | 0.90 | 0.02 | 0.78 |
| TPA and TPS | 0.35 | 0.08 | 0.70 | 0.0001 |

computer-aided scoring system based on multiple tests seems to be limited, and the use of several marker tests with similar antigenicity gives only little further benefit, whereas combinations of different kinds of markers may give more fruitful additional information. The best combination of these serum tumour marker tests in predicting pancreatic malignancy is the combination of CA 50 and TPA.

The authors wish to thank Mr Antero Julkunen BSc, Miss Raija Voutilainen BSc and Mr Tero Hongisto BSc for their assistance in the assay procedure. Special thanks go to Pharmacia Diagnostics, Uppsala, Sweden, for providing us with the CA 242 and CA 50 kits, to Beki Diagnostics, Bromma, Sweden, for providing us with the TPS kits and to Sangtec Medical, Bromma, Sweden, for providing us with the TPA kits used in this study.

### References

ALBERT, A. (1982). On the use and computation of likelihood ratios in clinical chemistry. *Clin. Chem.*, **28**, 1113-1139.

BENINI, L., CAVALLINI, G., ZORDAN, D., RIZZOTTI, P., RIGO, L., BROCCO, G., PEROBELLI, L., ZANCHETTA, M., PEDERZOLI, P. & SCURO, L.A. (1988). A clinical evaluation of monoclonal (CA 19-9, CA 50, CA 12-5) and polyclonal (CEA, TPA) antibody-defined antigens for the diagnosis of pancreatic cancer. *Pancreas*, **3**, 61-66.

BJÖRKLUND, B. (1980). On the nature and clinical use of tissue polypeptide antigen (TPA). *TumorDiagnostik*, **1**, 9-20.

BJÖRKLUND, B. & BJÖRKLUND, V. (1957). Antigenity of pooled human malignant and normal tissues by cyto-immunological technique: presence of an insoluble, heatlabile tumor antigen. *Int. Arch. Allergy*, **10**, 153-184.

BJÖRKLUND, B. & BJÖRKLUND, V. (1983). Specificity and basis of the tissue polypeptide antigen. *Cancer Detect. Prev.*, **6**, 41-50.

FEINSTEIN, A.R. (1985). *Clinical Epidemiology. The Architecture of Clinical Research.* W.B. Saunders: Philadelphia.

GOLDBERG, D.M. & ELLIS, G. (1978). Mathematical and computer-assisted procedures in the diagnosis of liver and biliary tract disorders. *Adv. Clin. Chem.*, **20**, 49-127.

HAGLUND, C. (1986). Tumour marker antigen CA 125 in pancreatic cancer: a comparison with CA 19-9 and CEA. *Br. J. Cancer*, **54**, 897-901.

HAGLUND, C., KUUSELA, P., JALANKO, H. & ROBERTS, P.J. (1987). Serum CA 50 as a tumour marker in pancreatic cancer: a comparison with CA 19-9. *Int. J. Cancer*, **39**, 477-481.

HAGLUND, C., LINDGREN, J., ROBERTS, P.J., KUUSELA, P. & NORDLING, S. (1989). Tissue expression of the tumour associated antigen CA 242 in benign and malignant pancreatic lesions. A comparison with CA 50 and CA 19-9. *Br. J. Cancer*, **60**, 845-851.

HAGLUND, C., ROBERTS, P.J., JALANKO, H. & KUUSELA, P. (1992). Tumour markers CA 19-9 and CA 50 in digestive tract malignancies. *Scand. J. Gastroenterol.*, **27**, 169-174.

KÖHLER, G. & MILSTEIN, C. (1975). Continuous cultures of fused cells secreting antibody predefined specificity. *Nature*, **256**, 495-497.

KUUSELA, P., HAGLUND, C. & ROBERTS, P.J. (1991). Comparison of a new tumour marker CA 242 with CA 19-9, CA 50 and carcinoembyronic antigen (CEA) in digestive tract disease. *Br. J. Cancer*, **63**, 636-640.

MASSON, P., PÅLSSON, B. & ANDREN-SANDBERG, Å. (1990). Cancer-associated tumour markers CA 19-9 and CA 50 in patients with pancreatic cancer with special reference to the Lewis blood cell status. *Br. J. Cancer*, **62**, 118-121.

PESANEN, P., PARTANEN, K., PIKKARAINEN, P., ALHAVA, E., PIRINEN, A. & JANATUINEN, E. (1991). Diagnostic accuracy of ultrasound, computed tomography and endoscopic retrograde cholangiopancreatography in the detection of obstructive jaundice. *Scand. J. Gastroenterol.*, **26**, 1157-1164.

PASANEN, P., ESKELINEN, M., PIKKARAINEN, P., ALHAVA, E., PARTANEN, K. & PENTTILÄ, I. (1992). Clinical evaluation of a new serum tumour marker CA 242 in pancreatic carcinoma. *Br. J. Cancer*, **65**, 731-734.

PASANEN, P., ESKELINEN, M., PARTANEN, K., PIKKARAINEN, P., PENTTILÄ, I. & ALHAVA, E. (1993). Receiver operating characteristic (ROC) curve analysis of the serum tumour markers CEA, CA 50 and CA 242 in the diagnosis of pancreatic cancer; results from a prospective study. *Br. J. Cancer*, **67**, 852-855.

TIAN, F., APPERT, H., MYLES, J. & HOWARD, J.M. (1992). Prognostic value of serum CA 19-9 levels in pancreatic adenocarcinoma. *Ann. Surg.*, **125**, 350-355.